# An exploration of maternity and newborn exposure, training and education among staff working within the North West Ambulance Service

**DOI:** 10.29045/14784726.2022.09.7.2.50

**Published:** 2022-09-01

**Authors:** Stephanie Heys, Susan Rhind, Joseph Tunn, Kate Shethwood, John Henry

**Affiliations:** North West Ambulance Service NHS Trust; University of Central Lancashire; North West Ambulance Service NHS Trust; North West Ambulance Service NHS Trust; North West Ambulance Service NHS Trust; Health Education England; North West Ambulance Service NHS Trust

**Keywords:** education, maternity, newborn, paramedic, pre-hospital, training

## Abstract

**Aim:**

Providing emergency and urgent care to pregnant patients and newborns in the pre-hospital setting often presents some of the most challenging and complex incidents attended to by ambulance staff. A service evaluation survey was undertaken to explore current levels of maternity and newborn education, preferred methods of training delivery, exposure and perceived support surrounding maternity and newborn care provision among pre-hospital clinicians working within the North West Ambulance Service (NWAS) NHS Trust.

**Methods:**

An online, anonymised survey compromising of 22 questions using multiple choice options and free-text questions was circulated among NWAS staff between 27 May 2021 and 21 June 2021. Questions explored the levels of training, education, exposure and confidence relating to maternal and newborn care in the pre-hospital setting. Insights into preferred approaches to engaging with continuing professional development (CPD) activities were also captured. Data were analysed using built-in Microsoft Forms analytics for quantitative response, with a basic thematic analysis undertaken to synthesise qualitative responses.

**Results:**

The survey received 509 responses, with data providing valuable insight relating to gaps in training provision, preferred approaches to CPD and barriers to engagement. Key themes focused on ‘pre-registration standards and variations’, ‘barriers and facilitators to continuing professional development’ and ‘exposure and skill decline: confidence and knowledge’.

**Conclusion:**

Areas for service improvement are highlighted, providing ambulance trusts and integrated care systems with key recommendations. These include maternity and newborn standards for education among paramedic science degree programmes; recognition of pre-hospital emergency maternity and newborn care among maternity providers; the need for exposure and regular multidisciplinary team (MDT) skills training for staff; and a collaborative system-led approach to scaling up and delivering MDT training that acknowledges pre-hospital clinicians as key care providers.

## Introduction

Despite maternity incidents making up less than 1% of calls attended to by the North West Ambulance Service (NWAS), pre-hospital maternity and newborn care provision requires attention. Maternity services are experiencing unprecedented demand, carrying cross-sectional consequences for the urgent and emergency care sector (Kinsman et al., 2020). Reducing unwarranted variation in experience or outcome is a focus of most trusts’ quality improvement strategies and a national driver for care delivery (Care Quality Commission, 2021; NHS England & NHS Improvement, 2020a). A recent national Healthcare Safety Investigations Branch report identified areas for improvement to enhance pre-hospital focus on subspecialities such as maternity care, recommending that the Association of Ambulance Chief Executives work together with the ambulance services to share best practice relating to the frequency and type of training pre-hospital clinicians receive to support standardisation and quality (Healthcare Safety Investigations Branch, 2021). Several national drivers in maternity care also focus on multidisciplinary team (MDT) training as a key recommendation to enhance safe, effective care delivery to women and babies across the United Kingdom (NHS England, 2019, 2020; Ockenden, 2020). However, a gap exists in how ambulance trusts are recognised and engaged with by maternity care providers as a key partner in emergency maternity and newborn care (McLelland et al., 2016), highlighting the need for a system-led approach to addressing such challenges. As part of the development of integrated care systems, a focus is placed on developing provider collaborations at scale (NHS England & NHS Improvement, 2020b), although at present the ambulance service is rarely acknowledged as a key provider of care within maternity safety agendas, locally and nationally. In NWAS, a system-level collaboration was agreed with the local maternity systems, for a fixed-term consultant midwife post.

## Background

Training for front line professionals has been identified as key to improving maternity care and safety (Draper et al., 2018; Knight et al., 2020; Merien et al., 2010; NHS England, 2019; NHS England & NHS Improvement, 2020b; NHS Litigation Authority, 2016; Widdows et al., 2018). Unplanned births in the pre-hospital setting are clinically significant events, which are often attended by ambulance crews (McClelland et al., 2019). Rapid clinical decision-making is often required when attending unplanned births in the community, emphasising the need for skilled and competent clinicians in attendance (Lazić & Takač, 2011; McClelland et al., 2019). The Better Births Agenda focused on enhancing multi-agency working to provide safe and effective maternity care within the United Kingdom (NHS England, 2016); however, regional variations persist regarding multi-agency approaches.

A recent review by the Health and Social Care Committee (2021) emphasises such points, noting that maternity services need to work collaboratively with key stakeholders, to enhance quality, governance and safety.

A lack of standardisation has meant that inconsistencies relating to the level of maternity and newborn education delivered to key care providers has been noted (Manktelow et al., 2016; NHS England, 2016, 2019; World Health Organization, 2010), acknowledging training as a key quality indicator for improving outcomes (Ockenden, 2020). It is known that an institutional knowledge gap exists in relation to maternity care provisions and training among those working within ambulance trusts (McLelland et al., 2013, 2016). Prior to 1999, education and training around obstetric care was not formally included within the paramedic curriculum (Woollard et al., 2010) and to date, higher education institutes (HEIs) have no standardised approach to the level, content and assessment strategies for obstetric and maternity training within the pre-registration paramedic science curriculum. Such gaps urge maternity systems to recognise the breadth, volume and types of maternity and newborn presentations attended to in the pre-hospital setting to address potential variation and gaps in current training approaches. Additionally, the current state of pre-hospital maternity education urges ambulance trusts to explore current maternity skills and training among staff working within the pre-hospital setting to identify areas of focus and quality improvement opportunities, supported by service evaluation approaches.

## Methods

This project was a service evaluation conducted through a staff survey. Service evaluations measure current practice and inform organisational improvement strategies (Healthcare Quality Improvement Partnership, 2020). The Checklist for Reporting Results of Internet E-Surveys (CHERRIES) was used to check the validity of the survey platform utilised to design and develop the survey and to mitigate as much bias as possible (Eysenbach, 2004).

### Study setting

NWAS is the ambulance service for north-west England and covers a population of approx. 7.5 million across Greater Manchester, Cheshire and Mersey, Lancashire and Cumbria. Based on 2020 data, NWAS employs 5544 ‘ambulance staff’ (North West Ambulance Service, 2020). Across the north-west, male and female life expectancy are both below the England average. The area includes high levels of social deprivation, with several local authorities within the north-west falling within the most deprived 10–20% in England (Office for National Statistics, 2021), highlighting the breadth of deprivation among the general population. Additionally, nine of the top 20 most deprived local authority areas are in the north-west of England (Office for National Statistics, 2022). The 2020 MBRRACE-UK (mothers and babies: reducing risk through audits and confidential enquiries across the UK) report highlights the complex interaction of risk factors for adverse outcomes in certain populations, including racial biases, complex life factors and existing conditions that place women and their babies at a higher risk of maternal mortality (Knight et al., 2020). Such statistics highlight the multi-faceted difficulties faced when delivering care in the pre-hospital setting and the multi-morbidity factors present among the pregnant population in the area.

Data collected from the NWAS maternity dashboard between 15 October 2020 and 15 October 2021 are presented in Table 1, providing an overview of maternity activity within the Trust.

**Table 1.** Maternity activity 15 October 2020 to 15 October 2021.

Emergency CAD records	n = 7743
Category 1	2300
Category 2	2164
Category 3	994
Category 4	95
Category 5	195
Hear and treat	9.8%
See and treat	15.5%
See and convey	74.8%

CAD: computer-aided dispatch.

### Data collection

Data were collected through a survey containing a mixture of 22 closed questions, to provide quantitative measurement, and open questions, to elicit qualitative insights (Hall, 2020). The survey aimed to elicit specific information relating to the perceived skills and confidence of staff; the level of maternity education and training they had received during pre-registration programmes; the level of continuing professional development (CPD) undertaken; and their exposure to maternity-related incidents. Additional questions focused on exploring preferences for training delivery platforms and suggestions on how best to engage in and access additional education and training resources focused on maternal and newborn care. The survey was distributed between 27 May 2021 and 21 June 2021 and was shared via internal communication channels at NWAS. The survey was open to all NWAS clinical staff and NWAS volunteers, with no exclusions. All responses were anonymous with no identifiable information collected. Participant information was provided to staff, including information on how responses would be summarised, analysed and shared, with consent gained prior to completion. Information was also included regarding the potential for responses to be published as part of a journal submission. The survey can be found at https://forms.office.com/Pages/ResponsePage.aspx?id=gpn262sDxEyyAnrrGUxQZb6g9TE7G9FEmo9i9QH_6eZUQjJGTFRGNUZIOFFGOUxCUFNDTTJaQkZVUy4u.

### Data analysis

Quantitative responses were collated and presented using the Microsoft Forms platform, which uses built-in analytics to present responses, and results were exported to Excel for additional analysis or grading. Descriptive data for qualitative responses were extracted, coded and grouped together by SH and SR and then analysed using a simple thematic approach by SH, SR, JT, KS in line with recent publications reporting analyses of narrative comments in postal questionnaires (Braun & Clarke, 2006; Downe et al., 2012; Redshaw & Henderson, 2012; Redshaw & Hockley, 2010; Thomson & Downe, 2016). Data were then presented in basic themes.

### Ethics and approvals

This project was registered and approved as a service evaluation by the NWAS Research and Development Department (Project Reference NWAS_EVAL_0032).

### Patient and public involvement

There was no patient or public involvement.

## Results

Overall, 509 members of staff completed the survey, representing 9.2% of estimated eligible clinical staff. 83% of respondents provided answers to each of the optional, multiple-choice questions. Responses are presented below under three thematic headings: maternity-related training and CPD; preferred format for and barriers to CPD and education; and exposure to maternity incidents, confidence and knowledge.

### Participant overview

The sample of respondents from each of the key NWAS regions was evenly split, with a 34% response rate from Cheshire and Mersey, 33% from Greater Manchester and 33% from Cumbria and Lancashire. The roles of those who responded are detailed in Table 2.

**Table 2.** Respondent role identification.

Role	(N = 509)	%
Paramedic	208	41
Senior paramedic / team leader	83	16
Emergency medical technician I	72	14
Newly qualified paramedic, year 1 and year 2	70	13
Advanced paramedic	18	4
Student paramedic	16	3
Emergency medical technician II	10	2
Emergency operations centre dispatcher	9	1.7
Operations manager	5	0.98
Critical care paramedic / HEMS	4	0.78
Allied health professional (nurse / midwife)	3	0.58
Enhanced / community first responder	2	0.39
Executive director	1	0.19
Ambulance care assistant	1	0.19

HEMS: helicopter emergency medical service.

### Maternity-related training and continuing professional development

The first section of the survey aimed to elicit information relating to pre- and post-registration training and education. Multiple-choice questions included ‘Did you receive any of the following during your clinical training or university/trust-specific education programme?’. Respondents could select more than one response. The results are presented in Table 3.

**Table 3.** Education and training identified.

Method (answers presented)	(N = 483)	%
Theory-based training relating to obstetric emergencies	395	82
Simulated training opportunities	236	49
Dedicated maternity-related module	197	41
Maternity-related OSCE	98	20
Formal exams relating to deterioration of the maternity patient	74	15

OSCE: objective structured clinical examination.

An additional question asked, ‘Have you attended or accessed any obstetric/maternity-related CPD events, i.e. podcasts / external training?’. 45% (n = 508) of all respondents stated that they had not accessed any obstetric/maternity-related CPD education or training. For the 54% (n = 508) who answered yes, the most cited platforms were:

- The College of Paramedics (Maternity CPD Day)
- Baby Lifeline Training
- The Resus Room Podcast
- Bespoke maternity events covering aspects of care that relate to paramedic scope of practice.

### Preferred format for and barriers to continuing professional development and education

Respondents’ preferences for platforms and training formats to undertake CPD were explored. Questions aimed to support the development of potential future resources and included ‘Please choose from the following options your preferred format of educational training / CPD’. Table 4 presents the responses and the preferred platforms in order of popularity. Respondents were able to choose more than one answer.

**Table 4.** Preferred format for continuing professional development and education.

Potential platforms and format of education delivery (answers presented)	(N = 405)	%
Face-to-face classroom teaching	400	99
Simulation/practical-based teaching	382	94
Trust-specific training days	287	71
Video format (pre-recorded)	184	45
Online seminar	148	37
Podcast	110	27
Case review learning	103	25
Written (books / training manuals)	100	24
College of Paramedics events	66	16
Ad hoc drop-in sessions by expert clinicians	65	16
Other	16	4

Despite over half of respondents stating they had accessed some form of training or educational resources, they noted this was done so in their own time. Only one respondent detailed placements within neonatal units or with neonatal teams. When provided the opportunity to add additional information, 38% (n = 301) highlighted the requirement for protected learning time to attend CPD.

### Exposure to maternity incidents, confidence and knowledge

The survey elicited feedback on experiences of incidents relating to maternity and neonatal cases to understand the level of exposure and chance to utilise learnt skills in practice. 48% (n = 507) of respondents had attended ‘fewer than 5’ maternity-related incidents in the past year. Respondents were asked to choose the most common presentations they have seen and managed within their role. Although less frequently attended, obstetric emergencies were also cited. See Table 5 for breakdown of the most common presentation attendance options provided.

**Table 5.** Most common presentation attendance options.

Presentation	(N = 498)	%
Birth imminent (normal birth)	357	72
Contractions (not in labour)	347	70
Established labour	204	41
Pre-term labour / pre-term birth	201	40
Antepartum bleeding (including antenatal haemorrhage)	187	38
Postnatal bleeding (including postpartum haemorrhage)	135	27
Unwell neonate (within 14 days of birth)	83	17
Newborn resuscitation	53	11
Breech	33	7
Maternal collapse	30	6
Cord prolapses	20	4
Shoulder dystocia	17	3

Key concerns in attendance at maternity-related calls were elicited via free-text responses. The top three concerns were: (1) a lack of preparation and knowledge to make informed clinical decisions; (2) not being able to access expert maternity support and advice; and (3) no standardised approach to providing pre-alerts to maternity units. When asked what they felt they needed to be competent in the management of maternity and newborn cases, 49% (n = 411) of respondents highlighted training to support skills and confidence; 31% (n = 411) cited placements; and 20% (n = 411) cited access to expert advice. Respondents were also asked ‘In your experience, can you detail any benefit of attending maternity-related calls?’, and provided with an optional free-text box. Of those that provided an answer, maternity incidents were seen to be rewarding, providing real-life experience and additional skills, and improving confidence.

## Discussion

Data collected via this service evaluation support the need for increased focus on maternity and newborn training within the ambulance service. Insights to preferred methods of training approaches among staff, and levels of engagement with educational resources have been identified.

A key theme highlighted within the data was the variation in standards and approaches in delivering maternity and newborn theory and skills across pre- and post-registration university- and trust-level education and training. Despite several respondents stating they had undertaken some level of maternity training, the type, frequency and approach varied. Standardised delivery of theory, skills and maternity placements across HEIs would support a reduction in variation. Hanna et al. (2021) explored experiences of learning, development and preparedness for clinical practice among undergraduate paramedicine students via a systematic review, and identified similar themes. The review highlighted a need for more effective communication between universities and integrated systems to address challenges in paramedicine education, supporting a collaborative identification of educational models that best facilitate learning, development and preparedness for clinical practice (Hanna et al., 2021). Despite a focus on pre-registration training and education, a gap exists in understanding of the level, depth and content of training provided to all staff who provide pre-hospital maternity and newborn care, including emergency medical technicians, community first responders and call takers.

Much of the CPD activity undertaken by staff was arranged by staff themselves in collaboration with maternity partners and often attended in staff members’ own time. A lack of governance and quality standards associated with accreditation of learning and acknowledgment of CPD for those who attend highlights a gap in the process to support and recognise staff engagement and is identified as a barrier within the literature among the paramedic profession (Gent, 2016; Hobbs et al., 2021). Just over half of respondents suggested trust-specific training days and/or paid learning and protected time as key to ensuring all staff attend and engage with training. Preference for training included face-to-face simulations and cross-disciplinary training, highlighted within a national study (Knox et al., 2014) and supported by the wider literature focused on obstetric-related skills acquisition (Jomeen et al., 2020). Additionally, several studies report increased confidence and skills among those attending MDT maternity training (Lenguerrand et al., 2020; Merien et al., 2010; Merriel et al., 2019). However, to date, the impact of such training on the skills and confidence of pre-hospital clinicians within UK ambulance trusts has not been measured, with previous work exploring pre-registration students’ experiences of training (Feltham et al., 2016; McLelland et al., 2013).

When attending incidents, respondents described concerns about a lack of knowledge; of access to expert clinicians to consult for advice and support; and of a clear process to support hospital alerts to receiving units. While a lack of confidence dealing with birthing-related emergencies has been identified within the literature (Dawson et al., 2003; Ledger et al., 2018), respondents noted that attending maternity and newborn cases increased experience, skills and confidence. Increased confidence following exposure to maternity care emergencies is also noted in the literature (Jomeen et al., 2020), supporting findings within this evaluation. The importance of building respectful relationships between providers to enhance working practices among multiple professions is also noted in the literature (Daelemans et al., 2021; NHS England, 2019; Ockenden, 2020), supporting the need for increased exposure for pre-hospital clinicians to gain experience within and alongside maternity colleagues.

### Service review recommendations

Insights gathered via this service evaluation place an increased emphasis on deploying skilled, competent and confident pre-hospital clinicians to address national agendas focused on safe, personal and quality-driven maternity and newborn care. Consequently, several recommendations, aimed at ambulance services and maternity system partners, are detailed as follows:

1. Standards for maternity and newborn training for pre-registration paramedics attending HEIs should be implemented to ensure equity and quality.
2. MDT training should acknowledge the context of the pre-hospital setting to ensure the scope of practice and the environment in which care is delivered is recognised among maternity professionals.
3. Regular exposure to childbirth, maternity complications and newborn care via CPD, simulations and maternity care placements should be supported among pre-hospital clinicians.
4. Standardised mandated maternity training and resources should be accessible to all pre-hospital clinicians to support the delivery of quality care to mothers and babies in the pre-hospital setting.

### Strengths and limitations

Limitations of closed questions are recognised when using survey methods to collect data, where further elaboration would have been useful. As the survey was only undertaken in one ambulance trust, transferability is limited. However, the results and insights presented as part of this service evaluation could serve as a benchmark for other organisations focusing on enhancing maternity and newborn training and education.

## Conclusion

This service evaluation has highlighted key areas for service improvement, focused on maternity and newborn care provision and training. Insights centre around collaborative working to enhance the skills and confidence of pre-hospital clinicians, with a focus on the need for integrated care systems to support standardisation and system-led approaches. Data also provide insights into preferences around training for staff and barriers to engagement. Providing pre-hospital clinicians with accessible, flexible and high-quality training and education aims to improve decision making and confidence when assessing and managing maternal and neonatal incidents. Several recommendations have been made aimed at ambulance services and maternity systems as a result of this service evaluation.

## Author contributions

SH, SR and JT were responsible for the design of the survey. SH co-ordinated data collection and analysis. SH, SR, JT and KS analysed the data. SR, KS and SH provided academic oversight throughout the service evaluation. All authors have read and approved the manuscript. We would like to acknowledge the important contribution of all those who completed the survey and thank research paramedic Betty Pennington and Professor Gill Thomson for proving a critical review. SH acts as the guarantor for this article.

## Conflict of interest

None declared.

## Ethics

Not required.

## Funding

None.
